# Cryptic Taxa Revealed through Combined Analysis of Chromosomes and DNA Barcodes: The *Polyommatus ripartii* Species Complex in Armenia and NW Iran †

**DOI:** 10.3390/insects15070545

**Published:** 2024-07-19

**Authors:** Vladimir A. Lukhtanov, Alexander V. Dantchenko

**Affiliations:** Department of Karyosystematics, Zoological Institute, Russian Academy of Sciences, Universitetskaya Nab. 1, 199034 Saint-Petersburg, Russia

**Keywords:** chromosome, *COI*, DNA barcode, karyotype, Lepidoptera, Lycaenidae, Polyommatinae, *Polyommatus*, sympatry

## Abstract

**Simple Summary:**

In the species-diverse butterfly genus *Polyommatus*, speciation is often driven by rapid changes in chromosome number and structure, resulting in cryptic species. These cryptic species are morphologically similar, but usually can be recognized relatively easily using chromosomal markers. In this work, we show that similar chromosome numbers can independently evolve in different phylogenetic lineages, resulting in species that are difficult to distinguish using routine cytogenetic techniques. We also demonstrate that the combined analysis of chromosomes and mitochondrial DNA barcodes is a simple and effective tool for identifying such cryptic species.

**Abstract:**

The detection of cryptic species in complexes that have undergone recent speciation is often difficult, since many standard nuclear markers have not yet accumulated differences between closely related taxa, and differences in mitochondrial markers can be leveled out due to mitochondrial introgressions. In these cases, the use of derived chromosomal characters such as non-ancestral chromosomal numbers and/or unusual karyotype features may be a solution to the species delimitation problem. However, non-ancestral but similar karyotypes may arise secondarily as a result of homoplastic evolution, and their interpretation as homologies may lead to incorrect taxonomic conclusions. In our study, we show that the combined use of mitochondrial DNA barcodes and karyotypes helps to solve this problem and identifies cryptic species in situations where each of these markers does not work individually. Using this approach, we show that the fauna of Armenia and adjacent Iran includes the following cryptic taxa of the *Polyommatus ripartii* species complex (haploid chromosome number, *n* in parentheses): *P. ripartii paralcestis* (*n* = 90), *P. ripartii kalashiani*, subsp. nov (*n* close to 90), *P. emmeli*, sp. nov. (*n* = 77–79), *P. keleybaricus*, sp. nov. (*n* = 86), *P. demavendi belovi* (*n* = 73–75), *P. demavendi antonius*, subsp. nov. (*n* = 71–73), *P. admetus anatoliensis* (*n* = 79) and *P. eriwanensis* (*n* = 29–34). *Polyommatus admetus yeranyani* is synonymized with *P. admetus anatoliensis*.

## 1. Introduction

Despite debates about species concepts and species criteria in biology [1], most scientists agree that speciation is complete when the divergence of evolutionary lineages results in their actual or potential ability to coexist in the same territory without mixing [2,3,4]. This ability to coexist is achieved through genetically determined physiological incompatibility, the separation of ecological niches, differences in reproductive behavior, or a combination of these mechanisms [5]. As a rule, species, even closely related ones, have fixed morphological differences, which arise either as a by-product of divergent evolution, or as an effect of the direct selection on increasing differences between species [5]. However, this is not always the case; species can be cryptic, that is, represent morphologically indistinguishable or highly similar biological entities [6,7,8,9,10,11,12,13,14,15].

Two different processes lead to the formation of cryptic species. Firstly, species are often formed in allopatry, when long-term evolution under conditions of geographic isolation results in postzygotically isolated, but morphologically similar lineages [16]. Secondly, speciation is often driven by the formation of prezygotic reproductive isolation, based on differences in ecology or behavior. However, the niche partitioning and recognition of conspecifics on the basis of non-visual mechanisms (for example, differences in acoustic signals and patterns of precopulatory behavior) do not necessarily promote morphological differentiation [17,18].

Genetic markers, such as DNA nucleotide substitutions and karyotype features, are the most reliable and universal characteristics used for the delimitation and identification of cryptic species [10,19,20,21,22]. Each of these two groups of markers has its own specifics, as well as its own advantages and disadvantages. Molecular markers, due to the more uniform dynamics of their evolutionary changes, are better suited to a general assessment of genetic divergence between taxa, which is important for the interpretation of the studied lineages in terms of different taxonomic ranks (genera, tribes, subfamilies, families, etc.) [23]. Chromosomal characteristics are often associated with (or even directly cause) postzygotic reproductive isolation. Therefore, they are more convincing as arguments for substantiating species-level taxonomic hypotheses [20,24]. It has long been believed that molecular markers (nucleotide substitutions) are more universal characteristics that can be used for substantiating taxonomic and phylogenetic hypotheses, since molecular differences between taxa are always present, and chromosomal differences are present only in limited numbers of cases [25]. However, this point of view can be considered outdated. Modern methods of karyotype analysis, based on chromosome-level genome assemblies, show that even homosequential species (i.e., species with identical karyotypes as identified by methods of light microscopy) differ in terms of multiple changes in the structure of their chromosomes. Therefore, de facto chromosomal rearrangements are universal characteristics used for solving problems of systematics and phylogenetics at all taxonomic levels, from the separation of closely related species and the description of subspecies to the analysis of phylogenetic relationships between phyla [26,27,28,29,30].

In real taxonomic practice, the possibilities of using molecular and chromosomal markers are limited by numerous technical, logistical and financial difficulties. If obtaining whole genome data and chromosome-level genome assemblies has become routine for species and populations from Western Europe, North America and East Asia [31,32], this is not so easy for the rest of the world. The delivery to a modern molecular laboratory of the fresh or freshly frozen (−80 °C) samples needed to obtain chromosome-level genome assemblies is practically impossible for numerous rare and undescribed species from Africa, South Asia and Siberia. Therefore, the simplest methods, such as DNA barcoding and routine karyotype analysis, remain the main avenues for obtaining primary genetic information about taxa. The combination of these two tools can have a strong synergistic effect [33,34].

In this study, we used the combined analysis of chromosomes and DNA barcodes to examine the taxonomic structures of blue butterflies of the so-called monomorphic complex, part of the mega-diverse genus *Polyommatus* Latreille, 1804 [35,36,37,38,39,40,41,42,43,44]. The complex received this name because, unlike other species of the genus, which are characterized by strong sexual dimorphism (males have blue wings; females have brown wings), both male and female representatives of the monomorphic complex have a similar brown color [33]. Almost all of the numerous species of this complex are cryptic taxa that are indistinguishable by coloration, wing pattern and genital structure, but can be distinguished by karyotypes and molecular markers [33,44,45].

Most species of this complex live in the southern part of the Western Palearctic, from Spain in the west to Iran in the east [45,46,47,48,49,50,51,52,53,54,55]. One species, *P. ripartii* (Freyer, 1830), has a wider distribution, covering the territories of Europe, Asia Minor, Central Asia, Southwest Siberia and West Mongolia [56,57,58,59]. Taxonomic revisions have recently been published relating to the species of this complex found in Western Europe [60], the Balkan Peninsula [61], Turkey [62] and Azerbaijan [33]. However, the taxa from the more eastern and southern territories remain poorly studied [33]. In this paper, based on collections and field studies made over 30 years, we present a taxonomic analysis of the species inhabiting Armenia and adjacent parts of Northwest Iran.

## 2. Materials and Methods

### 2.1. Analysis of Karyotype

Testes were removed from the male abdomens within 1 h of collection and were placed in a 3:1 fixative (3 parts 96% ethanol and 1 part glacial acetic acid). The testes were stored in this fixative for 1–4 weeks at 15–30 °C, and then for 1–10 years at −20 °C. The testes were stained in a saturated solution of orcein (Sigma, Steinheim, Germany) in 45% acetic acid for 7–20 days at 20 °C. We used a two-phase method of chromosome analysis, as described in [61]. First, the stained testes were placed in a 40% solution of lactic acid to soften them. The testicular membrane was ruptured using dissecting needles to remove the spermatocysts. The intact spematocysts with unsquashed metaphase plates were analyzed to count the numbers of bivalents in the first metaphase of meiosis or chromosomes in the second metaphase of meiosis. Then, the spermatocysts were squashed using light, gradually increasing pressure on the coverslip. This was achieved by pressing the index finger onto the coverslip and was monitored visually using a 20× and 40× microscope lens before applying immersion oil. This technique led to the fact that chromosomes and bivalents changed their position relative to each other, revealing cases of contact and overlap between chromosomes and bivalents.

The size (area) of bivalents was assessed visually (approximately, “by eye”). Although it is technically possible to calculate the area of bivalents in photographs in microns or pixels, such an “exact” calculation does not make sense for three reasons. First, the boundaries of bivalents in a microscope and in photographs are more of a blurred halo than an exact clear line. Second, the size of the bivalents depends to some extent on the saturation of the bivalents with the dye (orcein) during the staining process, and small chromosomes (bivalents) tend to swell more during the staining process than large chromosomes. The process of the saturation of bivalents with orcein is very difficult to control, since it depends on the quality of fixation of the material, which is different in the deep and superficial layers of the testis, and on the ages of the samples, which were different in our study. Third, the bivalent area is a projection onto the plane of a three-dimensional body with a complex configuration. It depends on the orientation of the bivalent in space. Despite these problems, it is clear from the chromosome preparations that in some cases the sizes of the bivalents differ markedly. Therefore, it was decided to evaluate the sizes of bivalents not quantitatively, but qualitatively, using four gradations: (1) the sizes are approximately the same, (2) one bivalent is approximately one and a half times (1.5 times) larger than the other, (3) one bivalent is larger than the other by more than one and a half times and less than two times (indicated as 1.5–1.8 times larger), and (4) one bivalent is twice as large.

A Leica DM2500 light microscope (Leica Microsystems, Wetzlar, Germany) equipped with an HC PL APO 100×/1.4 Oil CORR CS lens and S1/1.4 oil condenser head was used for bright-field microscopy analysis. The Leica lens HC PL APO 100×/1.40 Oil PH3 was used for the phase-contrast microscopy analysis.

### 2.2. Molecular Analysis

Standard mitochondrial DNA barcodes (658 bp COI gene fragments) were obtained for 64 samples (Appendix A) at the Canadian Center for DNA Barcoding (CCDB, Ontario Biodiversity Institute, University of Guelph) and at the Karyosystematics Department of the Zoological Institute of the Russian University, Academy of Sciences. The studied specimens are stored at the Zoological Institute of the Russian Academy of Sciences, St. Petersburg, Russia.

At the Canadian DNA Barcoding Centre, samples were processed according to the protocols described in [63,64,65]. At the Zoological Institute of the Russian Academy of Sciences, samples were processed according to the protocols described in [43,61]. For DNA amplification, standard mitochondrial barcode primers LepF1 and LepR1 were used [66,67]. All new COI sequences were deposited in GenBank.

The 64 *COI* sequences obtained in this study (Appendix A) and 63 published *COI* sequences representing the *P. ripartii* species complex and the outgroup (*P. icarus* (Rottemburg, 1775)) were aligned using the BioEdit software [68], resulting in 658 bp alignment (Supplementary File, Table S1). The Bayesian phylogenetic analysis was performed as previously described [61]. Briefly, the program MrBayes 3.2 [69] was used with default settings. Two runs of 10,000,000 generations with four chains (one cold and three heated) were performed. The first 25% of each run was discarded as burn-in. The consensus of the obtained trees was visualized using FigTree 1.4.4 (http://tree.bio.ed.ac.uk/software/), accessed on 18 October 2023). The minimum *COI* p-distances (%) between the taxa of the taxa were calculated using the MEGA 11 program [70].

### 2.3. Morphology

Butterfly photographs were taken with a Nikon D810 digital camera (Nikon Corporation, Minato City, Tokyo, Japan) equipped with a Nikon AF-S Micro Nikkor 105 mm lens, using the built-in flash as a lighting source.

## 3. Results

### 3.1. Karyotypes

**Figure 1:**
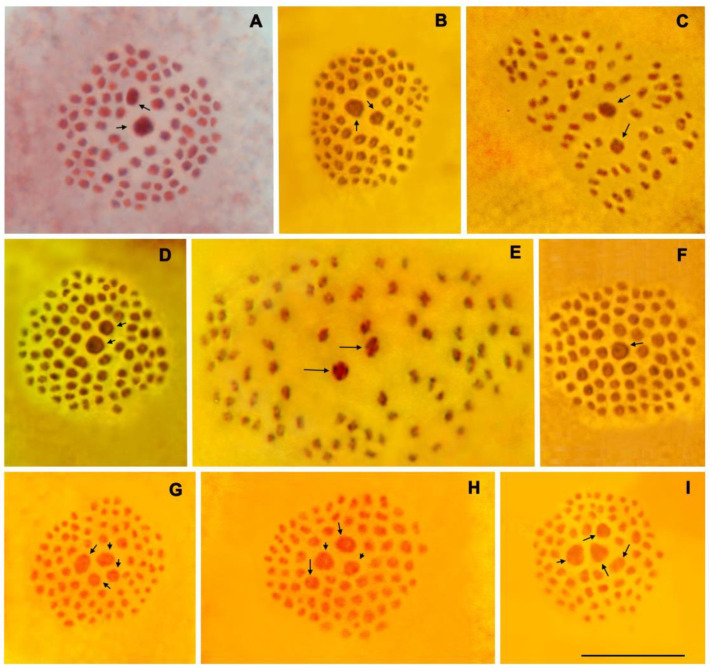
Karyotypes in the first metaphase of male meiosis. Arrows indicate large bivalents (two large bivalents in *P. ripartii*, *P. emmeli* and *P. keleybaricus*; one large bivalent in *P. admetus*, and four large bivalents in *P. demavendi*). (**A**) *P. ripartii*, sample 022K19, Tajikistan, *n* = 90. (**B**) *P. emmeli*, sample 320A08, Armenia, Gnishik, intact metaphase plate, *n* = 77. (**C**) *P. emmeli*, sample 318A08, Armenia, Gnishik, squash preparation, *n* = 78. (**D**) *P. keleybaricus*, sample E260, Iran, Keleybar, intact metaphase plate, *n* = 86. (**E**) *P. keleybaricus*, sample E262, Iran, Keleybar, holotype, squash preparation, *n* = 86. (**F**) *P. admetus anatoliensis*, sample E311, Iran, Azerbaijan-e Sharqi, Varzagan, intact metaphase plate, *n* = 79. (**G**) *P. demavendi belovi*, sample 121A07, Armenia, Vohkchaberd, intact metaphase plate, *n* = 75. (**H**) *P*. *demavendi belovi*, sample 054A07, Armenia, Khosrov, intact metaphase plate, *n* = 75. (**I**) *P. demavendi antonius*, sample 192A07, Armenia, Sevan Lake, intact metaphase plate, *n* = 71. Scale bar = 10 µm in all figures.

In this study, 48 specimens and eight taxa (species and subspecies) were karyotyped (Table 1, Figure 1). Additionally, 14 specimens of *P. eriwanensis* (Forster, 1960) karyotyped previously [71] were used for the analysis (Table 1). The chromosome numbers of all the taxa covered in this study, based on new data and previous results, are summarized in Table 2.

#### 3.1.1. *Polyommatus ripartii* (Figure 1A)

At the MI stage, 90 chromosome bivalents were observed in the studied specimen 022K19 from Tajikistan. Of these, two bivalents can be classified as very large bivalent 1 and medium-sized bivalent 2. The largest first bivalent was approximately 1.5–1.8 times larger than the medium-sized bivalent 2. The smaller bivalents, from 3 to 90, were found to form a gradient size row in which the largest element 3 was approximately two to three times larger than the smallest element. The largest bivalents 1 and 2 were always observed in the center of metaphase plates.

A similar karyotype was found in samples from Armenia (Gyumarants and Ja Joor pass), but the number of chromosomes was determined to be approximately equal to 90, due to the fact that it was not possible to find metaphase plates of ideal quality without overlapping and touching chromosomes.

#### 3.1.2. *Polyommatus emmeli*, sp. nov. (Figure 1B,C)

A variable number (from *n* = 77 to *n* = 79) of distinct chromosome elements were found at the MI and MII cells of the studied 14 specimens. In structure (the number of large bivalents and their relative sizes), the karyotype was similar to that of *P. ripartii*. There are two large elements in the set. At the MI stage, the first and largest bivalent was approximately 1.5–1.8 times larger than the medium-sized bivalent 2. The remaining smaller bivalents were found to form a gradient size row in which the largest element 3 was approximately two to three times larger than the smallest element. The largest bivalents 1 and 2 were always observed in the center of metaphase plates.

#### 3.1.3. *Polyommatus keleybaricus*, sp. nov. (Figure 1D,E)

The haploid chromosome number was identified to be *n* = 86 based on analyses of five samples (Table 1). In terms of the structure of the MI metaphase plates (the number of large bivalents and their relative sizes), the karyotypewas similar to that of *P. ripartii*. There are two large elements in the set. At the MI stage, the largest first bivalent was approximately 1.5–1.8 times larger than the medium-sized bivalent 2. The remaining smaller bivalents were found to form a gradient size row in which the largest element 3 was approximately two to three times larger than the smallest element. The largest bivalents 1 and 2 were always observed in the center of metaphase plates.

#### 3.1.4. *Polyommatus admetus yeranyani* (Dantchenko et Lukhtanov, 2004) (Figure 1F)

The haploid chromosome number was identified to be *n* = 79 based on analyses of five samples (Table 1). In the sample KL-34-96 from Armenia (Aragats), the haploid chromosome number was identified to be close to *n* = 80; however, this count was made with an approximation due to the fact that it was not possible to find metaphase plates of ideal quality without overlapping and touching chromosomes. Bivalents at the MI stage and chromosomes at the MII stage are highly differentiated in size, and one of the largest element, located in the center, is always easily recognized. All other elements, from medium to very small, form a gradient size row, within which the individual identification of chromosomes is impossible. Thus, this karyotype differs from that of *P. emmeli*, which also has a haploid chromosome number of *n* = 79.

#### 3.1.5. *Polyommatus demavendi belovi* (Dantchenko et Lukhtanov, 2004) (Figure 1G,H)

A variable number (from *n* = 73 to *n* = 75) of distinct chromosome elements were found at the MI and MII cells of the studied 12 specimens. In terms of structure (the number of large bivalents and their relative sizes), the karyotype is distinct if compared with karyotypes of *P. ripartii*, *P. emmeli*, *P. keleybaricus* and *P. admetus*. There are two large and two medium-sized elements in the set. At the MI stage, the two largest bivalents were approximately 1.5 times larger than the two medium-sized bivalents. The remaining smaller bivalents were found to form a gradient size row. The two largest bivalents 1 and 2 and the two medium-sized bivalents 3 and 4 were always observed in the center of metaphase plates.

#### 3.1.6. *Polyommatus demavendi antonius*, subsp. nov. (Figure 1I)

A variable number (from *n* = 71 to *n* = ca73) of distinct chromosome elements were found at the MI and MII cells of the six studied specimens. In terms of structure (the number of large bivalents and their relative sizes), the karyotypeis similar to that of *Polyommatus demavendi belovi*. There are two large and two medium-sized elements in the set. At the MI stage, the two largest bivalents were approximately 1.5 times larger than the two medium-sized bivalents. The remaining smaller bivalents were found to form a gradient size row. The two largest bivalents 1 and 2 and the two medium-sized bivalents 3 and 4 were always observed in the centers of metaphase plates.

### 3.2. Phylogenetic Analysis and Clustering of the Studied Samples by COI Haplotypes and Karyotypes

**Figure 2:**
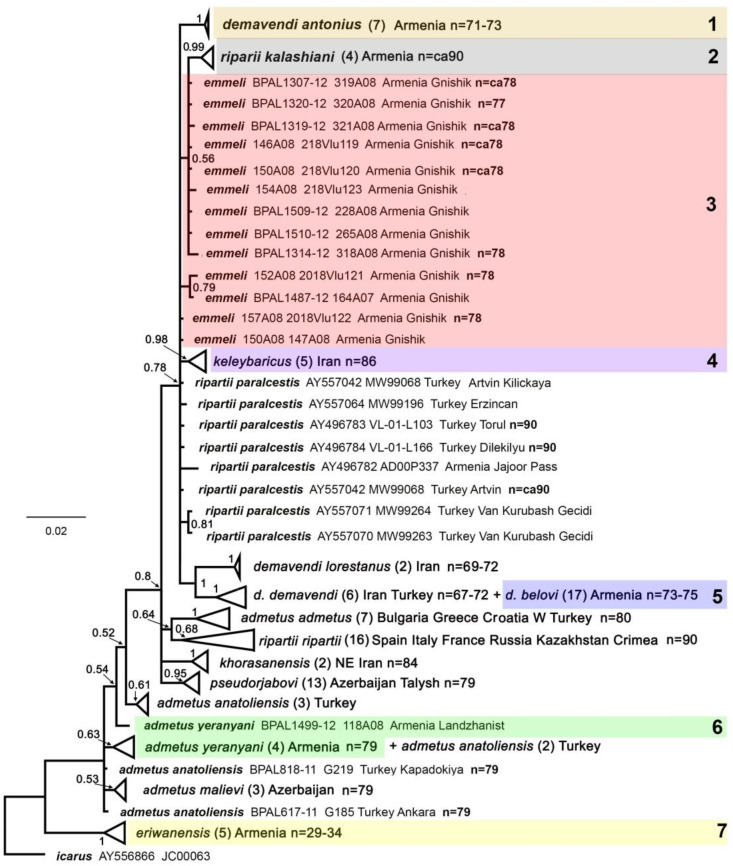
The Bayesian tree based on analysis of the mitochondrial *COI* barcodes. Numbers at nodes indicate Bayesian posterior probabilities (BPP) (higher than 0.5). The number in brackets (after species and subspecies names) refers to the number of analyzed specimens. 1–7 are differentiated groups of individuals found in Armenia and the adjacent territory of Iran (highlighted by different colors). The groups 3, 5 and 6 are paraphyletic. Samples from other territories are not highlighted. Scale bar = 0.02 substitutions per position. Groups of individuals with support higher than 0.5 are collapsed to make the Figure 2 more compact. The finer structure of the collapsed groups is shown in Figure S1 in Supplementary Materials.

The Bayesian analysis (Figure 2) identified *P. eriwanensis* as a highly differentiated clade, sister to the lineage containing the remaining taxa. Within the latter lineage, specimens of *P. admetus* (Esper, 1783) from eastern Turkey (*P. admetus anatoliensis* (Forster, 1960)), Iran, Armenia (*P. admetus yeranyani* (Dantchenko et Lukhtanov, 2004)), and Azerbaijan (*P. admetus malievi* (Dantchenko et Lukhtanov, 2004)) formed a basal polytomy. Specimens of *P. admetus admetus* from southeastern Europe and western Turkey were identified as a discrete lineage differentiated from *P. admetus anatoliensis* (including *P. admetus yeranyani*) that appeared on the tree as a paraphyletic assemblage. In should be noted that the posterior supports for this paraphyletic topology were low and not inconsistent with the monophyly of *P. admetus anatoliensis* if markers with stronger phylogenetic signal are used.

The taxa *P. khorasanensis* (Carbonell, 2001) (*n* = 84) (Table 2) and *P. pseudorjabovi* Lukhtanov, Dantchenko, Vishnevskaya, Saifitdinova, 2015 (*n* = 79) (Table 2) were identified as strongly supported lineages. The remaining samples formed a weakly supported cluster, which included a polytomy consisting of samples of *P. ripartii paralcestis* (Forster, 1960) and *P. emmeli*, as well as the supported sublineages *P. demavendi antonius*, *P. ripartii kalashiani*, *P. keleybaricus*, *P. demavendi lorestanus* Eckweiler, 1997 and (*P. demavendi demavendi* (Pfeiffer, 1938) + *P. d. belovi*).

The studied samples from Armenia and the adjacent territory of Iran (highlighted by colors on the phylogeny in Figure 2) were found to form seven differentiated groups of individuals. Four of these groups (1, 2, 4 and 7) are highly supported monophyletic lineages. The groups 3, 5 and 6 appeared on the DNA barcode tree as paraphyletic assemblages, although their monophyly cannot be excluded and could theoretically be justified in the future using markers with a stronger phylogenetic signal.

Group 1 (*P. demavendi antonius*) is monophyletic with respect to the *COI* gene. This group is characterized by haploid chromosome numbers *n* = 71–73; the set contains four very large bivalents. This group is similar in karyotype to *P. demavendi demavendi* (*n* = 67–72), *P. demavendi lorestanus* (*n* = 69–72) and *P. demevendi amasyensis* (*n* = 70–72) (Table 2).

Group 2 (*P. ripartii kalashiani*) is monophyletic with respect to the *COI* gene. This group is characterized by a haploid number of chromosomes *n* = ca90; in the set there is one large and one medium bivalent. This group has a karyotype indistinguishable from that of *P. ripartii paralcestis* and *P. ripartii ripartii* (Table 2).

Group 3 (*P. emmeli*) has DNA barcodes indistinguishable from those of *P. riparti paralcestis*, but this group has a unique karyotype distinctly different from the karyotype of *P. ripartii*. It is characterized by haploid chromosome numbers *n* = 77–79; the set contains one large and one medium bivalent, distinguishing it from *P. riparti paralcestis* (*n* = 90) (Table 2).

Group 4 (*P. keleybaricus*) is monophyletic with respect to the *COI* gene. It has a unique karyotype (*n* = 86) (Figure 1D, E).

Group 5 (*P. demavendi belovi*) has DNA barcodes indistinguishable from those of *P. demavendi demavendi* from Iran and *P. demavendi amasyensis* from Turkey, but has a higher number of chromosomes than *P. demavendi demavendi* and *P. demavendi amasyensis* (Table 2).

Group 6 (*P. admenus yeranyani*) is indistinguishable by karyotypes (Table 2) and DNA barcodes (Figure 2) from *P. admetus anatoliensis*.

Group 7 (*P. eriwanensis*) is monophyletic with respect to the *COI* gene. It is characterized by haploid chromosome numbers *n* = 29–34 (Table 1 and Table 2).

It should be noted that representatives of the groups 3 (*P. emmeli*), 5 (*P. demavendi belovi*), 6 (*P. admetus yeranyani*) and 7 (*P. eriwanensis*) were found in sympatry in Gnishik (Table 1).

### 3.3. Color and Wing Pattern

**Figure 3:**
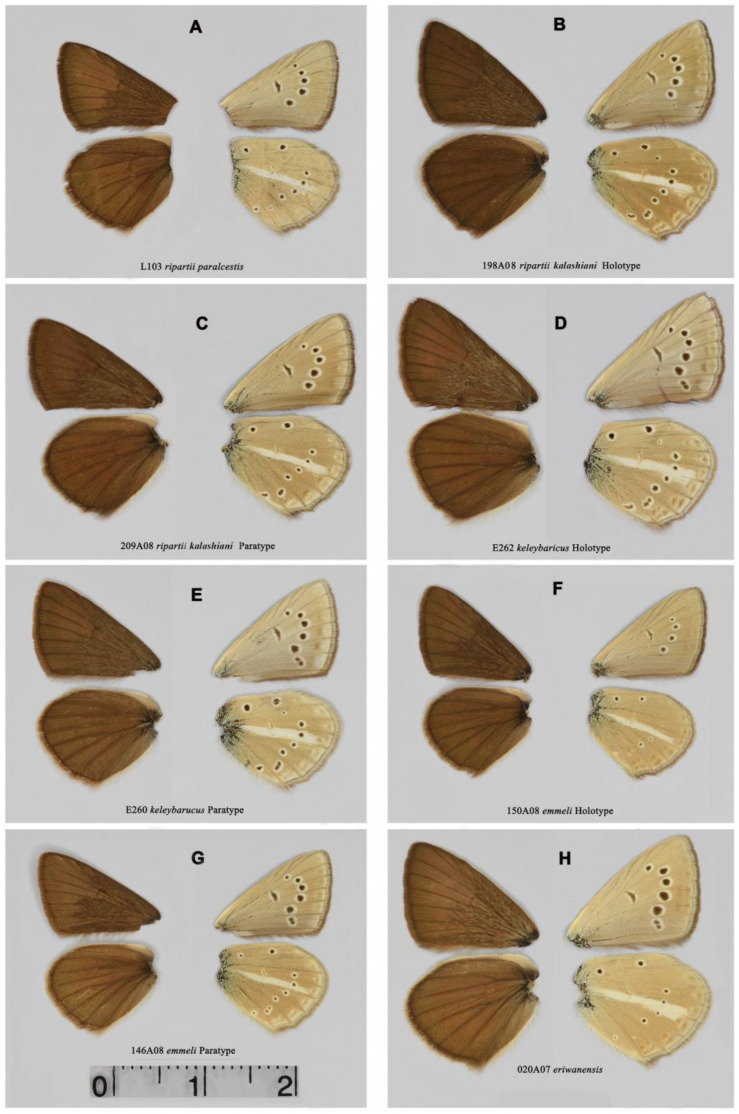
Upperside (**left**) and underside (**right**) of the male wings. (**A**) *P. ripartii paralcestis*, sample L103, Turkey. (**B**) *P. ripartii kalashiani*, ssp. nov., sample 198A08, holotype, Armenia, Shvanidzor, Gyumarants. (**C**) *P. ripartii kalashiani*, ssp. nov., sample 209A08, paratype, Armenia, Shvanidzor, Gyumarants. (**D**) *P. keleybaricus*, sp. nov., sample E262, holotype, Iran, Keleybar. (**E**) *P. keleybaricus*, sp. nov., sample E260, paratype, Iran, Keleybar. (**F**) *P. emmeli*, sp. nov., sample 150A08, holotype, Armenia, Gnishik. (**G**) *P. emmeli*, sp. nov., sample 146A08, paratype, Armenia, Gnishik. (**H**) *P. eriwanensis*, sample 020A07, Armenia, Gnishik. The scale bar (2 cm) applies to all wings.

**Figure 4:**
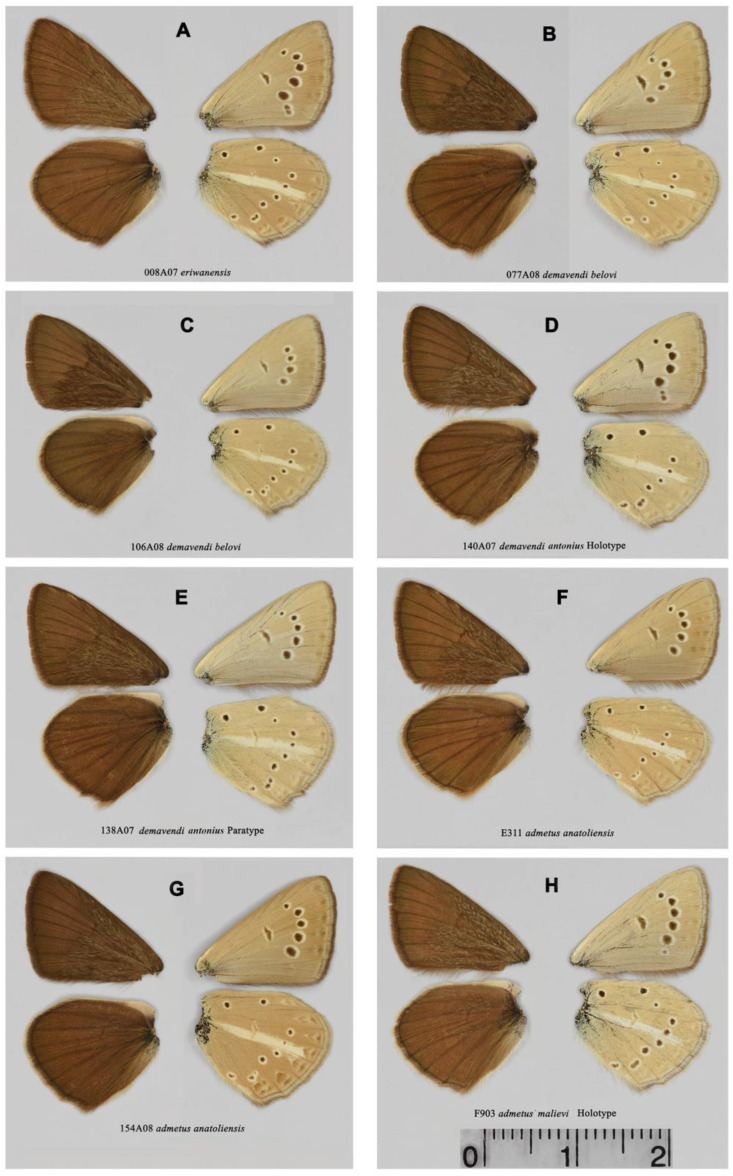
Upperside (**left**) and underside (**right**) of the male wings. (**A**) *P. eriwanensis*, sample 008A07, Armenia, Gnishik. (**B**) *P.demavendi belovi*, sample 077A08, Armenia, Gnishik. (**C**) *P. demavendi belovi*, sample 106A08, Armenia, Khosrov. (**D**) *P. demavendi antonius*, ssp. nov., sample 140A07, holotype, Armenia, Sevan Lake. (**E**) *P. demavendi antonius*, ssp. nov., sample 138A07, paratype, Armenia, Sevan Lake. (**F**) *P. admetus anatoliensis*, sample E311, Iran, Varzagan. (**G**) *P. admetus anatoliensis* (yeranyani), sample 154A08, Armenia, Gnishik. (**H**) *P. admetus malievi*, sample F903, holotype, Azerbaijan, Talysh. The scale bar (2 cm) applies to all wings.

All studied butterflies are indistinguishable by the upper side of the wings, which have a dark brown color in males with a dense network of androconial scales (Figure 3 and Figure 4). The underside is characterized by the presence of a white stripe on the hind wings and a system of basal, discal and postdiscal spots, as well as a marginal pattern, the intensity of which varies to the greatest extent from almost complete reduction (Figure 3A) to a high degree of intensity Figure 3B–E and Figure 4G). A more detailed analysis of this figure is given below when describing the species and subspecies.

## 4. Discussion

Butterflies of the studied *P. ripartii* complex from Armenia and Iran are among the so-called cryptic species, the identification of which is possible when based on the study of chromosomal and molecular markers, but impossible when based on morphology [33,36,39,46,47,48,49,50,51,52,53,54,55,56,57,58,59,60,61]. Within these butterflies, our molecular and chromosomal analyses revealed several genetic clusters, of which four (*emmeli*, *keleybaricus*, *antonius* and *kalashiani*) were not recognized previously.

It would be tempting to treat all these clusters as taxa, but discrete genetic differences between individuals are not yet evidence that they belong to different species [23]. Groups of individuals, discrete by genetic markers, may represent either different taxa, or variants of intraspecific genetic polymorphism. We previously proposed general principles for the interpretation of such clusters, based on the joint use of karyotype characteristics and DNA barcodes [33]. The chromosomal characters and the mitochondrial DNA barcodes represent two different, not physically linked parts of the genome, namely, nuclear (karyotypes) and mitochondrial (DNA barcodes) markers. In the case of intra-population variability, the linkage equilibrium of nuclear and mitochondrial markers is expected [34]. This indicates the situation in which the markers combine at random and form all possible combinations of mitochondrial and chromosomal characteristics. Additionally, in the case of intra-population variability, we should expect numerous chromosomal heterozygotes that can be recognized via tri- and multivalents observed at the first meiotic metaphase [20,33,61]. In the case of two cryptic species, hybridization between the clusters is absent or very limited. Therefore, we should expect a situation in which nuclear and mitochondrial genes mimic linkage disequilibrium, forming stable species-specific combinations [33]. In addition, in the case of cryptic species, the absence of interspecific hybridization leads to the absence of heterozygotes for chromosomal rearrangements [20,33,61].

**Figure 5:**
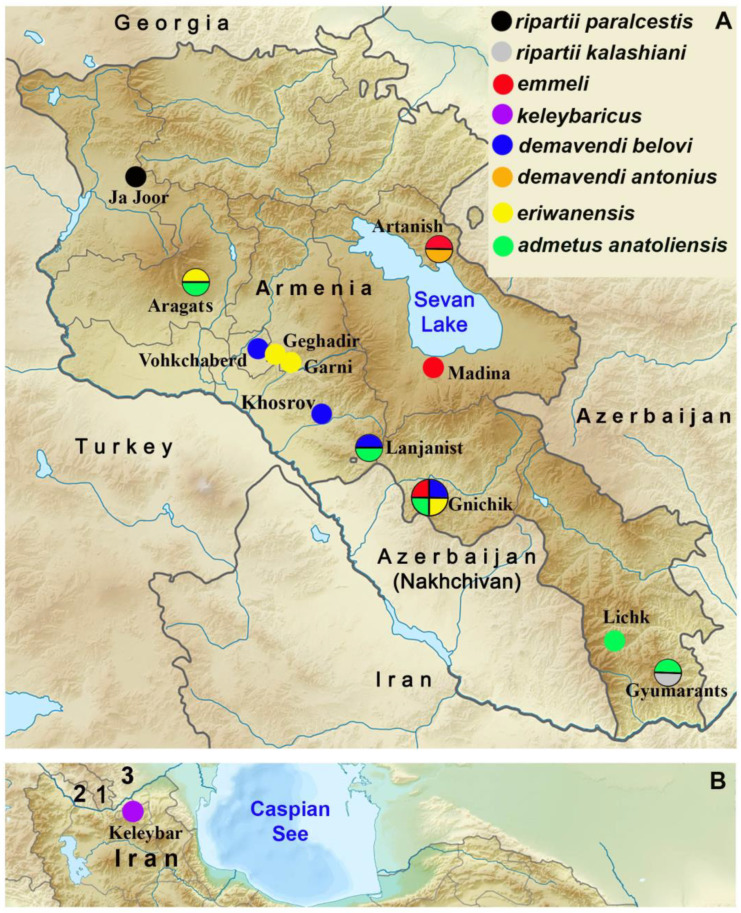
Maps of Armenia (**A**) and North Iran (**B**) showing the distribution of *P. ripartii paralcestis* (black), *P. ripartii kalashiani* (grey), *P. emmeli* (red), *P. keleybaricus* (violet), *P. demavendi belovi* (blue), *P. demavendi antonius* (brown), *P. eriwanensis* (yellow), and *P. admetus anatoliensis* (green). In (**B**), 1 is Armenia, 2 is Azerbaijan (Nakhchivan), and 3 is Azerbaijan. The size of the circle corresponds to the number of species found in the locality (one, two or four species).

In our study, the latter situation was found in Gnishik (south slopes of Vayots Dzor range, Armenia), where we discovered four clusters (*eriwanensis*, *admetus*, *demavendi belovi* and *emmeli*) in complete sympatry (Figure 5). Of these four clusters, three (*eriwanensis, admetus*, and *demavendi belovi*) were described previously as taxa [33,71], and the fourth cluster is described in this work below as a new species, *P. emmeli*, sp. nov. Within these four sympatric clusters, each entity is characterized by a stable combination of a specific mitochondrial haplogroup and a unique karyotype. During our study, we also did not find chromosomal heterozygotes that are expected to exist in the case of interspecific hybridization. All this indicates that all four clusters, including the previously unknown *P*. *emmeli*, belong to different biological species.

When the clusters are allopatric, the direct application of the species criteria based on the limited interspecific hybridization is not possible. Therefore, two groups of individuals living in allopatry should be considered different species if they are differentiated so strongly that reproductive isolation between them can be assumed, or if their merging would result in non-monophyletic assemblage [33,34]. The presence of fixed chromosomal rearrangements between two or more groups of individuals, as a rule, indicates their non-conspecificity [20,44,45,59,60,61], since usually even relatively small differences in karyotypes lead to disorders of meiosis and hybrid sterility [72]. Nevertheless, in some cases, differences in karyotypes do not lead to postzygotic isolation [73]. In particular, in Lepidoptera, there are cases of fertility in hybrids between chromosomally divergent races [73], as well as high intraspecific variability in the number of chromosomes [44,45]. The latter phenomenon is incompatible with strong selection against chromosomal heterozygotes. However, it should be borne in mind that even in this case, chromosomal differences affect reproductive isolation, suppressing recombination in rearranged chromosome regions in hybrids [74,75,76,77,78].

The criterion of chromosomal differences can be used for the taxonomic interpretation of the allopatric taxa *P. riparii paralcestis* (*n* = 90), *P. emmeli* (*n* = 77–79) and *P. keleybaricus* (*n* = 86), which differ in chromosomal numbers. All these three taxa have similar mitochondrial DNA barcodes, which indicates either the origin of these taxa from a common ancestor, or relatively recent mitochondrial introgression. A comparative phylogenetic analysis of the accumulation of chromosomal rearrangements leading to changes in chromosome numbers in the genus *Polyommatus* shows that chromosome numbers evolve slowly, and there is a significant correlation between chromosome number changes and phylogeny branch lengths [79]. Therefore, given that mitochondrial introgression is common in different organisms, including Lepidoptera [80,81,82,83,84,85,86,87], the DNA barcode identity between deeply differentiated chromosomal lineages appears to be a consequence of mitochondrial introgression rather than the ancestrality of this character. This hypothesis should be tested in future via analyses of nuclear markers that are expected to be differentiated. Therefore, we believe that fixed differences between the karyotypes of *P. riparii paralcestis* (*n* = 90), *P. emmeli* (*n* = 77–79) and *P. keleybaricus* (*n* = 86) allow us to interpret them as different species.

The allopatric taxa *P. demavendi demavendi*, *P. demavendi amasiensis*, *P. demavendi lorestanus* and *P. demavendi belovi* [33,88], and the population from the Lake Sevan region, which will be described below as *P. demavendi antonius*, have similar chromosome numbers and an identical karyotype structure (a combination of two large and two medium chromosomes), which can be interpreted as a chromosomal synapomorphy. On this basis, they can be combined into one taxon of species rank (*P. demavendi*), despite the fact that the monophyly of this group is unresolved on the tree based on its short DNA barcodes.

Within this species, two subspecies (*P. d. lorestanus* and *P. d. antonius*) are well differentiated by mitochondrial DNA and represent distinct phylogenetic sublineages. As for *P. d. belovi* and *P. d. demavendi*, they show differences in chromosome numbers, which are close and variable within each group (*n* = 67–72 in *P. d. demavendi* and *n* = 73–75 in *P. d. belovi*), but do not overlap [33,88]. Thus, the combined molecular and chromosomal data provide evidence of the subspecies status of *P. demavendi demavendi*, *P. demavendi lorestanus, P. demavendi belovi* and *P. demavendi antonius*.

Another subspecies, *P. demavendi amasyensis*, is found in central Turkey [39,40,41]. According to the available data on karyotypes (88), it is indistinguishable from *P. demavendi demavendi*. Therefore, we tentatively consider it as a synonym of *P. demavendi demavendi*. Thus, the species *P. demavendi* includes four subspecies: (1) *P. demavendi antonius* (Lake Sevan region in Armenia), (2) *P. demavendi belovi* (southern part of Armenia), *P. demavendi demavendi* (=*amasyensis*) (northwestern Iran, eastern and central Turkey), and (4) *P. demavendi lorestanus* northern and central Zagros in Iran).

The allopatric taxa *P. ripartii paralcestis* and *P. ripartii kalashiani* are also differentiated by DNA barcodes, and have clear differences in the wing color. These taxa can be interpreted as subspecies, if by subspecies we mean differentiated phylogeographic sublineages [89].

In our study, we found a lack of differentiation by DNA barcodes between *P. admetus anatoliensis* (central Turkey) and *P. admetus yeranyani* (Armenia). The karyotypes of these two taxa are also identical. Thus, these nominal taxa do not represent phylogeographic sublineages, which is essential for justifying subspecific status [89]. As for morphology, butterflies with a pronounced marginal pattern on the underside of the hindwings predominate in central Turkey. In Armenia and adjacent regions of Iran, butterflies with this phenotype are also found (Figure 4G); however, individuals with a reduced marginal pattern predominate (Figure 4F). Thus, no fixed differences were found between populations from central Turkey and Armenia. On this basis, the taxon *P. admetus yeranyani*, syn nov. is synonymized here with *P. admetus anatoliensis*.

The subspecies *P. admetus malievi*, found in the easternmost part of the species’ range, also does not differ from *P. admetus anatoliensis* in karyotype [33]. Its monophyly for the *COI* gene has not been proven in our study due to the low support for the cluster formed by individuals of this subspecies. However, the difference in phenotypes between *P. admetus malievi* and *P. admetus anatoliensis* is substantial, and we do not have material showing the clinal morphological transition from *P. admetus malievi* to *P. admetus anatoliensis*. On this basis, we propose to maintain the subspecies status of *P. admetus malievi*.

## 5. Description of New Taxa

*Polyommatus emmeli* Dantchenko et Lukhtanov, sp. nov.

HOLOTYPE. Male. Field code 150A08, GenBank accession no. PP600665. Armenia, Vayots Dzor Province, Areni Municipality, vicinity of Gnishik (Armenian: Գնիշիկ), 39.673° N, 45.291° E, 2000 m, 19 July 2008, A.V. Dantchenko leg. In Zoological Institute of the Russian Academy of Science (St. Petersburg).

PARATYPES. Thirty-six males. One male, field code KL-49-97, same locality and collector as holotype, but June 1997. One male, field code 183A07, same locality and collector as holotype, but July 2007. Fifteen males, field codes 146A08, 150A08, 152A08, 157A08, 158A08, 242A08, 257A08, 261A08, 264A08, 302A08, 318A08, 319A08, 320A08, 321A08, 326A08 same locality and collector as holotype, but 19–30 July 2008. Nine males, field codes, 077K16A, 083K16A, 088K16A, 093K16A, 095K16A, 098K16A, 105K16A, 11K16A, 113K16A, same locality and collector as holotype, but 28 July 2016. One male, field code 234K16A, Hermon (Armenian: Հերմոն), Yeghegis Municipality of the Vayots Dzor Province, 39.902°N, 45.442°E, 1950 m, 18 July 2016, A.V.Dantchenko leg. One male, field code 243K16A, Armenia, Gegharkunik Province, Sevan Lake (south), Martuni Municipality, vicinity of Madina (Armenian: Մադինա), 40.472° N, 45.257° E, 2147 m, 22 August 2016, A.V. Dantchenko leg. Ten males, field codes 404-406K15A, 408-410K15A, 412K15A, 417-419K15A, Armenia, Gegharkunik Province, Shoghakat Municipality, South slopes of Artanish mnt. (Armenian: Արտանիշ), 40.472° N, 45.317° E, 2100 m, 19 July 2015, A.V. Dantchenko leg. Seven males, field codes 584K15A, 586K15A, 587K15A, 589-591K15A, 595K15A, same locality, but 23 July 2015, A.V. Dantchenko leg. All paratypes are preserved in the Zoological Institute of the Russian Academy of Science (St. Petersburg, Russia).

Males (Figure 3F,G). Forewing length in holotype 15.1 mm. Forewing length in paratypes 14.9–16.8 mm.

Upperside. Ground color is dark brown with slightly darker veins. Discoidal, submarginal and antemarginal marking is absent on both fore- and hindwings. Forewings have good developed sex brands and scaletufts. Fringe is brown, as is ground color.

Underside. Ground color is light greyish brown. Greenish blue basal suffusion on the hindwings is slightly visible. Discoidal black spots are present on forewings. Postdiscal black marking is small or even reduced on hindwings and or medium-sized on fore wings, circled with white on both fore- and hindwings. Submarginal markings are well pronounced on the hindwing, antemarginal markings are presented by fuzzy lunulae. On the forewings, sub- and antemarginal markings are strongly reduced, practically absent. White streak on hindwings underside clearly visible, enlarged distally. Fringe light brown, slightly darker than underside ground color.

Genitalia. In males, the genitalia have the same structure as in other species of the *Polyommatus* subgenus *Agrodiaetus* Hübner, 1822 [90]. We did not find any species-specific features in their structure. Briefly described, it can be noted that the uncus consists of two sclerotized parts. Gnathos has the appearance of sclerotized hooks. Yuxta has two long narrow branches. The relatively short aedeagus is straight, not curved. The lengths of the valvae are more than four times their width. There is a convex membranous fold on the ventral surface of the valva. The sacculus is well developed and extends along the entire ventral margin of the valvae.

Females. Although we have collected a significant number of females that are highly likely to belong to this species, we choose not to describe them here for the following reasons. Reliable identification of the species using DNA barcodes is impossible, since it shares barcodes with *P. ripartii*. *Polyommatus ripartii* is not found in Gnishik and Sevan, where the new species is described; however, it is found in adjacent territories, and we cannot completely exclude its sympatry with *P. emmeli*. The reliable identification of *P. emmeli* is possible by karyotype, but methods for analyzing female karyotypes for the genus *Polyommatus* have not been developed. Therefore, the description of females will only be possible after they are collected in copula with males, if the karyotype of these males is studied.

Karyotype. See Results section above (Table 1, Figure 1B,C).

Diagnosis. *Polyommatus emmeli* can be reliably differentiated from other species by its chromosome number of *n* = 77–79, in combination with the presence of two marker chromosomes, the largest in the set and the middle one, which is noticeably smaller than the first, but substantially larger than the third and subsequent ones in the set. It differs from the sympatric *P. admetus*, which also has *n* = 79, by its karyotype structure (*P. admetus* has only one large chromosome in the set) and DNA barcodes. It differs from the allopatric *P. pseudorjabovi* (*n* = 79) by the structure of the karyotype (*P. pseudorjabovi* has four large chromosomes in the set) and fixed nucleotide substitutions in DNA barcodes.

Phenotypically, it differs from the sympatric species *P. demavendi*, *P. admetus* and *P. eriwanensis* by its smaller average size, more pointed forewings and a tendency to decrease or even reduce the pattern elements on the underside of the hindwings. However, it should be noted that in terms of wing pattern, there is overlap between the sympatric species *P. emmeli*, *P. admetus*, *P. demavendi* and *P. eriwanensis*, and identification using these characters is unreliable.

Bionomy. *Polyommatus emmeli* inhabits tragacanth steppes and dry meadows, mainly on southern slopes at an altitude of 1850 to 2200 m above sea level. It was found in sympatry with *P. demavendi belovi*, *P. admetus anatoliensis* and *P. eriwanensis* in the Vayots Dzor mountains. In 1997, *P. emmeli* was collected in the form of last-instar larvae from a distinctive *Onobrychis* species (Fabaceae), which was described later [91] as *Onobrychis takhtajanii* Sytin, 2000. Most probably, this *Onobrychis* species is a food plant for *Polyommatus emmeli*.

Distribution. Armenia: Vayots Dzor range and the mountains surrounding Lake Sevan (Figure 5A).

Etymology. Named in honor of Thomas Emmel (1941–2018), a famous American entomologist, one of the initiators of cytogenetic studies of butterflies in the Caucasus and Transcaucasia.

*Polyommatus keleybaricus* Dantchenko et Lukhtanov, sp. nov.

HOLOTYPE. Male. Field code E262, BOLD # BPAL588-11, GenBank accession no. PP600681. Iran, East Azerbaijan Province (Azarbaijan-e Sharqi), Kaleybar County, Makidi vill. (W of Kaleybar), 38.85° N, 46.89° E, 1700–2000m, 21 July 2004, A.V. Dantchenko & V.A. Lukhtanov leg. In Zoological Institute of the Russian Academy of Science (St. Petersburg, Russia).

PARATYPES. Seven males. Field codes E250, E258, E260 (GenBank EF104628), E261, VL790 (GenBank PP600682), VL791 (GenBank PP600683) and VL792 (GenBank PP600684), the same locality, date and collectors as holotype. All paratypes are preserved in the Zoological Institute of the Russian Academy of Science (St. Petersburg, Russia).

Males (Figure 3D,E). Forewing length in holotype 18.1 mm. Forewing length in paratypes 16.5–17.5 mm.

Upperside. Ground color is dark brown with slightly darker veins. Discoidal, submarginal and antemarginal markings are absent on both fore- and hindwings. Forewings have good developed sex brands and scaletufts. Fringe is brown, as is ground color. In general, the upperside of the wings is not different from that of *P. emmeli*.

Underside. Ground color is light greyish brown. Greenish blue basal suffusion on the hind wings is slightly visible. Basal black spots are present only on hindwings. Discoidal black spots are present on forewings. Postdiscal black spots are relatively large on both fore- and hindwings, circled with white on both fore- and hindwings. Submarginal markings are very well pronounced on hindwings, and antemarginal markings are also present. On the forewings, the sub- and antemarginal markings are less pronounced than on the hindwing, but are clearly present. The white streaks on the hindwings’ undersides are bright, and enlarged distally. Fringe light brown, slightly darker than underside ground color.

Genitalia. The male genitalia have a structure typical for other species of the subgenus *Agrodiaetus* [90].

Karyotype. See Results section above (Table 1, Figure 1D,E).

Females. Unknown. No females with the *keleybaricus* mitochondrial haplogroup were found within the studied samples.

Diagnosis. *Polyommatus keleybaricus* (*n* = 86) reliably differ from the closest allopatric species *P. ripartii* (*n* = 90) and *P. emmeli* (*n* = 77–79) in terms of the number of chromosomes and by fixed nucleotide substitution T⇔A in the position 411 of the studied *COI* region. It is reliably distinguished from all other species by its karyotype: the set contains *n* = 86 in combination with the presence of two marker chromosomes, the largest in the set and the middle one, which is noticeably smaller than the first, but significantly larger than the third and subsequent ones in the set. In addition, the new species differs from all species except *P. ripartii* and *P. emmeli* by multiple nucleotide substitutions in the mitochondrial DNA barcode. Phenotypically, it differs from all other taxa (except *P. ripartii kalashiani*) by the strong lightening of the marginal region on the underside of the fore- and hindwings, against which a contrasting marginal pattern is developed. *Polyommatus ripartii kalashiani* has a similar type of pattern on the underside of the wings; however, the wings of *P. ripartii kalashiani* have a darker brownish color on the underside.

Bionomy. *Polyommatus keleybaricus* inhabits meadows and clearings on borders of mountain oak forests. The biotope where it lives is relatively humid and is characterized by the presence of mesophilic species of boreal and Hyrcanian origin (e.g., *Argynnis alexandra* Ménétriès, 1832).

Distribution. Found only in the type locality (Figure 5B).

Etymology. The name is toponymic. Keleybar (Kaleybar, Kalibar) is a city in the Central District of Keleybar County, East Azerbaijan province, Iran, serving as the capital of both the county and the district.

*Polyommatus demavendi antonius* Dantchenko et Lukhtanov, subsp. nov.

HOLOTYPE. Male. Field code 140A07, GenBank accession no. PP600657. Armenia, Gegharkunik Province, Sevan Lake, Artanish Municipality, South slopes of Artanish mnt., 40.468° N, 45.306° E, July 2007, A.V. Dantchenko leg. In Zoological Institute of the Russian Academy of Science (St. Petersburg, Russia).

PARATYPES. Five males, field codes 138A07, 151A07, 184A07, 192A07, 195A07, the same locality, date and collectors as holotype. Seven males, field codes 362-368K15A, the same locality, but 19 July 2015. Five males, field codes 402K14A, 407K15A, 415K15A, 422K15A, 423K15A, the same locality but 23 July 2015. All paratypes are preserved in the Zoological Institute of the Russian Academy of Science (St. Petersburg, Russia).

Males (Figure 4D,E). Forewing length in holotype 17.4 mm. Forewing length in paratypes 17.0–18.0 mm.

Upperside. Ground color is dark brown with slightly darker veins. Discoidal, submarginal and antemarginal markings are absent on both fore- and hindwings. Forewings have good developed sex brands and scaletufts. Fringe is brown, as is ground color. In general, the uppersides of the wings are not different from those of *P. emmeli* and *P. keleybaricus*.

Underside: ground color light brown with grayish tint. Greenish blue basal suffusion is slightly visible. Discoidal black spots present on forewings. Postdiscal black marking small on hindwings and/or medium sized on forewings, sharply contrasted by the grayish ground color on both fore- and hindwings. Submarginal marking reduced on hindwing, antemarginal markings are presented by fuzzy spots. On the forewings, the sub- and antemarginal markings are strongly reduced and slightly pronounced as diffused spots. The white streak on the hindwings’ underside is long, usually not enlarged distally. Fringe is light brown, slightly darker than the underside ground color.

Genitalia. The male genitalia have a structure typical for other species of the subgenus *Agrodiaetus* [90].

Karyotype. See Results section above (Table 1, Figure 1I).

Females. Unknown. No females with the *keleybaricus* mitochondrial haplogroup were found within the studied samples.

Diagnosis. *P. demavendi antonius* differs from other subspecies of *P. demavendi* by a set of fixed nucleotide substitutions in mitochondrial DNA barcodes and a lighter, milky coffee color with grayish tint on the underside of the wings. From *P. demavendi belovi*, which occurs in close proximity in the more southern parts of Armenia, the new subspecies differs in the large number of chromosomes, indicating the presence of one or even two fixed chromosomal fusions/fissions separating these subspecies.

Bionomy. *Polyommatus demavendi antonius* inhabits xerophilic calcerous slopes with tragacanth vegetation in the juniper woodland belt from 2100 to 2400 m above sea level. The last-instar larvae were collected from the end of May until the middle of June on *Onobrychis radiata* Bieberstein, 1810 (Section *Heliobrychis*), which is most probably a larval food plant for *P. demavendi antonius*. The butterflies are on their wings from the end of June. Ecologically, *P. demavendi antonius* differs from *P. demavendi belovi*, which inhabits dry biotopes often on gypsum soils in lowland desert-like areas. The larval food plant of *P. demavendi belovi* is *Onobrychis atropatana* Boiss.

Distribution. Northern Armenia (Lake Sevan region) (Figure 5A).

Etymology. Named in honor of Anton Dantchenko, who helped in collecting and fixing the butterflies on our trip to Lake Sevan.

*Polyommatus ripartii kalashiani* Dantchenko et Lukhtanov, subsp. nov.

HOLOTYPE. Male. Field code 198A08. Armenia, Syunik Province, Meghri Municipality, Shvanidzor (Armenian: Շվանիձոր), Gyumarants, 39.003° N, 46.380° E, 1722 m, 22 July 2008, A.V. Dantchenko leg. In Zoological Institute of the Russian Academy of Science (St. Petersburg, Russia).

PARATYPES. Twenty-five males with field codes 181A08, 182A08, 183A08, 184A08, 185A08, 186A08, 187A08, 189A08, 190A08, 191A08, 191F08C, 193A08, 194A08, 195A08, 196A08 (GenBank #PP600685), 197A08 (GenBank #PP600686), 201A08, 202 A08 (GenBank #PP600687), 203A08 (GenBank #PP600688), 204A08, 205A08, 206A08, 207A08, 209A08, and 210A08, the same locality and collector as holotype, 21–22 July 2008. One male, Dan2001-13, the same locality and collector, July 2001.

Males (Figure 3B,C). The forewing length in the holotype is 16.9 mm. The forewing length in paratypes is 16.5–17.0 mm.

Upperside. The ground color is dark brown with slightly darker veins. Discoidal, submarginal and antemarginal markings are absent on both fore- and hindwings. Forewings have good developed sex brands and scaletufts. The fringe is brown, as is the ground color. In general, the uppersides of the wings are no different from those of the other studied taxa.

Underside. Ground color is greyish brown. Greenish blue basal suffusion on the hindwings is clearly visible. Basal black spots are present only on hindwings. Discoidal black spots are present on forewings. Postdiscal black spots are relatively large on both fore- and hindwings, circled with white on both fore- and hindwings. The submarginal marking is very well pronounced on the hindwing, and the antemarginal marking is also present. On the forewings, sub- and antemarginal markings are less pronounced than on the forewing, but are nevertheless clearly present. The white streak on the hindwings’ underside is bright, enlarged distally. Fringe is light brown, slightly darker than underside ground color.

Genitalia: the male genitalia have a structure typical for other species of the subgenus *Agrodiaetus* [90].

Karyotype. See Results section above (Table 1).

Females. Unknown. No females with the *keleybaricus* mitochondrial haplogroup were found within the studied samples.

Diagnosis. *Polyommatus ripartii kalashiani* differs from other subspecies of *P. ripartii* in the strong lightening of the marginal region on the undersides of the fore- and hindwings, against which a contrasting marginal pattern is developed. *P. keleybaricus* has a similar type of pattern on the underside of the wings; however, in *P. keleybaricus*, the wings have a lighter gray-brownish color on the underside. It differs from species *P. ripartii paralcestis, P. keleybaricus* and *P. emmeli* by the fixed nucleotide substitution A⇔G in the position 420 of the studied *COI* region.

Bionomy. *Polyommatus ripartii kalashiani* inhabits dry meadows and clearings on the upper borders of mountain oak forests, from 1400 to 1900 m above sea level. The butterflies are on their wings from mid-July to August. The larvae’s food plant is, most probably, an undetermined *Onobrychis* species (section *Onobrychis*) [92], which is common in the biotope.

Distribution. Southern Armenia (Syunik Province), south slopes of Zangezur mountains (Figure 5A).

Etymology. Named in honor of Mark Kalashian, our friend and colleague, a prominent Armenian zoologist.

## Figures and Tables

**Table 1:** List of the karyotyped samples with their chromosome numbers.

Species	Lab Id	GenBank#	Chromosome Number	Locality	Reference
*P. emmeli*	KL-49-97		*n* = 79	Armenia, Gnishik	This study
*P. emmeli*	183A07		*n* = ca79	Armenia, Sevan Lake	This study
*P. emmeli*	146A08	PP600664	*n* = ca78	Armenia, Gnishik	This study
*P. emmeli* Holotype	150A08	PP600665	*n* = 78	Armenia, Gnishik	This study
*P. emmeli*	152A08	PP600666	*n* = 78	Armenia, Gnishik	This study
*P. emmeli*	157A08	PP600667	*n* = 78	Armenia, Gnishik	This study
*P. emmeli*	158A08		*n* = 79	Armenia, Gnishik	This study
*P. emmeli*	318A08	PP600670	*n* = 78	Armenia, Gnishik	This study
*P. emmeli*	319A08	PP600669	*n* = ca78	Armenia, Gnishik	This study
*P. emmeli*	320A08	PP600672	*n* = 77	Armenia, Gnishik	This study
*P. emmeli*	321A08	PP600671	*n* = ca77	Armenia, Gnishik	This study
*P. emmeli*	234K16A		*n* = 77	Armenia, Vayots Dzor	This study
*P. emmeli*	243K16A		*n* = 77	Armenia, Sevan Lake, Madina	This study
*P. emmeli*	584K15		*n* = 77	Armenia, Sevan Lake, Artanish	This study
*P*. *ripartii ripartii*	022K19		*n* = 90	Tajikistan, Jirgatol	This study
*P*. *ripartiiparalcestis*	Dan2001-13		*n* = ca90	Armenia, Ja Joor pass	This study
*P. ripartii kalashiani* Holotype	198A08		*n* = ca90	Armenia, Shvanidzor	This study
*P. ripartii kalashiani*	201A08		*n* = ca90	Armenia, Shvanidzor	This study
*P. ripartii kalashiani*	209A08		*n* = ca90	Armenia, Shvanidzor	This study
*P. keleybaricus*	E250		*n* = ca86	Iran, Keleybar, Makidi	This study
*P. keleybaricus*	E258		*n* = ca86	Iran, Keleybar, Makidi	This study
*P. keleybaricus*	E260	EF104628	*n* = 86	Iran, Keleybar, Makidi	This study
*P. keleybaricus*	E261		*n* = ca86	Iran, Keleybar, Makidi	This study
*P. keleybaricus* Holotype	E262	PP600681	*n* = 86	Iran, Keleybar, Makidi	This study
*P. admetus*	E311		*n* = 79	Iran, Azerbaijan-e Sharqi, Varzagan	This study
*P. admetus yeranyani*	KL-34-96		*n* = ca80	Armenia, Aragats	This study
*P. admetus yeranyani*	KL-50-97		*n* = 79	Armenia, Aragats	This study
*P. admetus yeranyani*	KL-67-97		*n* = 79	Armenia, Megri, Lichk	This study
*P. admetus yeranyani*	154A08	PP600630	*n* = 79	Armenia, Gnishik	This study
*P. admetus yeranyani*	211A08		*n* = 79	Armenia, Gyumarants	This study
*P. demavendi belovi*	KL-28-97		*n* = 74	Armenia, Khosrov	This study
*P. demavendi belovi*	003A07		*n* = 73	Armenia, Gnishik	This study
*P. demavendi belovi*	050A07		*n* = 74	Armenia, Khosrov	This study
*P. demavendi belovi*	051A07		*n* = 74	Armenia, Khosrov	This study
*P. demavendi belovi*	054A07		*n* = 74	Armenia, Khosrov	This study
*P. demavendi belovi*	064A07		*n* = 73	Armenia, Khosrov	This study
*P. demavendi belovi*	070A07		*n* = 73	Armenia, Khosrov	This study
*P. demavendi belovi*	077A08		*n* = 74	Armenia, Gnishik	This study
*P. demavendi belovi*	079A08		*n* = 74	Armenia, Gnishik	This study
*P. demavendi belovi*	106A08		*n* = 73	Armenia, Khosrov	This study
*P. demavendi belovi*	121A07		*n* = 75	Armenia, Vohkchaberd	This study
*P. demavendi belovi*	2002Q479		*n* = ca73	Armenia, Khosrov	This study
*P. d. antonius*	138A07		*n* = ca72	Armenia, Sevan Lake	This study
*P. d. antonius* Holotype	140A07	PP600657	*n* = 71	Armenia, Sevan Lake	This study
*P. d. antonius*	151A07		*n* = 71	Armenia, Sevan Lake	This study
*P. d. antonius*	184A07	PP600658	*n* = ca73	Armenia, Sevan Lake	This study
*P. d. antonius*	192A07		*n* = 71	Armenia, Sevan Lake	This study
*P. d. antonius*	195A07		*n* = ca71	Armenia, Sevan Lake	This study
*P. eriwanensis*	KL-1996-34-1		ca32	Armenia, Aragats	[71]
*P. eriwanensis*	KL-1997-6-1		ca34	Armenia, Garny	[71]
*P. eriwanensis*	KL-1997-6-4		*n* = ca31	Armenia, Garny	[71]
*P. eriwanensis*	KL-1997-6-7		*n* = 34	Armenia, Garny	[71]
*P. eriwanensis*	KL-1997-6-8		*n* = ca34	Armenia, Garny	[71]
*P. eriwanensis*	KL-1997-6-9		*n* = 33	Armenia, Garny	[71]
*P. eriwanensis*	KL-1997-7		*n* = 29	Armenia, Garny	[71]
*P. eriwanensis*	KL-1997-76-1		*n* = 34	Armenia, Gnishik	[71]
*P. eriwanensis*	AD2001-Nr4		*n* = ca30	Armenia, Geghadir	[71]
*P. eriwanensis*	AD2001-008		*n* = 34	Armenia, Gnishik	[71]
*P. eriwanensis*	001A07		*n* = 34	Armenia, Gnishik	[71]
*P. eriwanensis*	002A07		*n* = 32	Armenia, Gnishik	[71]
*P. eriwanensis*	004A07		*n* = 32	Armenia, Gnishik	[71]
*P. eriwanensis*	004A09		*n* = ca32	Armenia, Gnishik	[71]

**Table 2:** Chromosome numbers of all the taxa covered in this study, based on new data and previous results.

Taxa	Chromosome Number	Distribution Range	Reference
*P. admetus admetus*	*n* = 80	Balkan Peninsula, West Turkey	[45,61]
*P. admetus anatoliensis*	*n* = 78–79	East Turkey	[45]
*P. admetus malievi*	*n* = 78–79	Azerbaijan	[33]
*P. admetus yeranyani*	*n* = 79	Armenia	This study
*P. demavendi amasyensis* (de Lesse, 1961)	*n* = 70–72	Northern Central Turkey	[45]
*P*. *demavendiantonius*	*n* = 71–73	North Armenia	This study
*P. demavendi belovi*	*n* = 73–75	Armenia	This study
*P. demavendi demavendi*	*n* = 67–72	North Iran, East Turkey	[45]
*P. demavendi lorestanus*	*n* = 69–72	Iran (North and Central Zagros)	[45]
*P. emmeli*	*n* = 77–79	Armenia	This study
*P. eriwanensis*	*n* = 29–34	Armenia	[71]
*P. keleybaricus*	*n* = 86	Northwest Iran	This study
*P. khorasanensis*	*n* = 84	Northeast Iran	[33]
*P. pseudorjabovi*	*n* = 79	Azerbaijan	[33]
*P. ripartii kalashiani*	*n* = ca90	Southeast Armenia	This study
*P. ripartii paralcestis*	*n* = 90	East Turkey, West Armenia	[45]
*P. ripartii ripartii*	*n* = 90	from Spain to Mongolia and Central Asia	[45,59,60,61], this study

## Data Availability

All the analyzed DNA sequences are available via the GenBank links provided.

## Supplementary Materials

The following supporting information can be downloaded at: https://www.mdpi.com/article/10.3390/insects15070545/s1, Table S1. The 658 bp *COI* alignment analyzed in this study. Figure S1. The Bayesian tree of all studied samples based on analysis of the mitochondrial *COI* barcodes (uncollapsed tree). Numbers at nodes indicate Bayesian posterior probabilities (BPP) (higher than 0.5). 1–7 are differentiated groups of individuals found in Armenia and the adjacent territory of Iran (highlighted by different colors). Samples from other territories are not highlighted. Scale bar = 0.02 substitutions per position.

## Appendix A. List of the Studied Samples of the Genus Polyommatus and Obtained COI Sequences

**Species**	**BOLD/** **Field Id**	**GenBank ID**	**Chromosome Number**	**Country**	**Locality**
*P. admetus anatoliensis*	BPAL617-11/G185	PP600625	*n* = 79	Turkey	Ankara
*P. admetus anatoliensis*	BPAL618-11/G219	PP600626	*n* = 79	Turkey	Cappadocia
*P. admetus anatoliensis*	BPAL705-11	PP600627		Turkey	Kelkit
*P. admetus anatoliensis*	BPAL707-11	PP600628		Turkey	Tercan
*P. admetus anatoliensis*	BPAL708-11	PP600629		Turkey	Tercan
*P. admetus yeranyani*	BPAL1330-12/154A08	PP600630	*n* = 79	Armenia	Gnishik
*P. admetus yeranyani*	BPAL1471-12/017A07	PP600631		Armenia	Gnishik
*P. admetus yeranyani*	BPAL1475-12/021A07	PP600632		Armenia	Gnishik
*P. admetus yeranyani*	BPAL1476-12/025A07	PP600633		Armenia	Gnishik
*P. admetus yeranyani*	BPAL1477-12/026A07	PP600634		Armenia	Gnishik
*P. admetus yeranyani*	BPAL1495-12/083A08	PP600635		Armenia	Gnishik
*P. admetus yeranyani*	BPAL1497-12/086A08	PP600636		Armenia	Gnishik
*P. admetus yeranyani*	BPAL1499-12/118A08	PP600637		Armenia	Landzhanist
*P. admetus yeranyani*	BPAL1501-12/148A08	PP600638		Armenia	Gnishik
*P. admetus yeranyani*	BPAL1503-12/155A08	PP600639		Armenia	Gnishik
*P. demavendi belovi*	2018VLu112/003A07	PP600640	*n* = 73	Armenia	Gnishik
*P. demavendi belovi*	2018Vlu113/050A07	PP600641	*n* = 74	Armenia	Khosrov
*P. demavendi belovi*	2018Vlu114/121A07	PP600642	*n* = 75	Armenia	Vokhchaberd
*P. demavendi belovi*	2018Vlu115/105A08	PP600643		Armenia	Khosrov
*P. demavendi belovi*	2018Vlu116/106A08	PP600644	*n* = 73	Armenia	Khosrov
*P. demavendi belovi*	BPAL1308-12/077A08	PP600645	*n* = 73–74	Armenia	Gnishik
*P. demavendi belovi*	BPAL1309-12/079A08	PP600646	*n* = 73–74	Armenia	Gnishik
*P. demavendi belovi*	BPAL1478-12/049A07	PP600647		Armenia	Khosrov
*P. demavendi belovi*	BPAL1479-12/052A07	PP600648		Armenia	Khosrov
*P. demavendi belovi*	BPAL1480-12/055A07	PP600649		Armenia	Khosrov
*P. demavendi belovi*	BPAL1481-12/119A07	PP600650		Armenia	Vokhchaberd
*P. demavendi belovi*	BPAL1482-12/120A07	PP600651		Armenia	Vokhchaberd
*P. demavendi belovi*	BPAL1492-12/002A08	PP600652		Armenia	Khosrov
*P. demavendi belovi*	BPAL1493-12/010A08	PP600653		Armenia	Khosrov
*P. demavendi belovi*	BPAL1494-12/011A08	PP600654		Armenia	Khosrov
*P. demavendi belovi*	BPAL1496-12/084A08	PP600655		Armenia	Gnishik
*P. demavendi belovi*	BPAL1498-12/107A08	PP600656		Armenia	Khosrov-Agasi
*P. demavendi antonius* Holotype	2018Vlu117/140A07	PP600657	*n* = 71	Armenia	Sevan
*P. demavendi antonius*	2018Vlu118/184A07	PP600658	*n* = 73	Armenia	Sevan
*P. demavendi antonius*	BPAL1483-12/137A07	PP600659		Armenia	Sevan
*P. demavendi antonius*	BPAL1484-12/139A07	PP600660		Armenia	Sevan
*P. demavendi antonius*	BPAL1485-12/156A07	PP600661		Armenia	Sevan
*P. demavendi antonius*	BPAL1486-12/158A07	PP600662		Armenia	Sevan
*P. demavendi antonius*	BPAL1488-12/190A07	PP600663		Armenia	Sevan
*P. emmeli*	2018Vlu119/146A08	PP600664	nca78	Armenia	Gnishik
*P. emmeli* Holotype	2018Vlu120/150A08	PP600665	*n* = 78	Armenia	Gnishik
*P. emmeli*	2018Vlu121/152A08	PP600666	*n* = 78	Armenia	Gnishik
*P. emmeli*	2018Vlu122/157A08	PP600667	*n* = 78	Armenia	Gnishik
*P. emmeli*	2018Vlu123/154A08	PP600668		Armenia	Gnishik
*P. emmeli*	BPAL1307-12/319A08	PP600669	nca78	Armenia	Gnishik
*P. emmeli*	BPAL1314-12/318A08	PP600670	*n* = 78	Armenia	Gnishik
*P. emmeli*	BPAL1319-12/321A08	PP600671	nca78	Armenia	Gnishik
*P. emmeli*	BPAL1320-12/320A08	PP600672	*n* = 77	Armenia	Gnishik
*P. emmeli*	BPAL1487-12/164A07	PP600673		Armenia	Gnishik
*P. emmeli*	BPAL1500-12/147A08	PP600674		Armenia	Gnishik
*P. emmeli*	BPAL1509-12/228A08	PP600675		Armenia	Gnishik
*P. emmeli*	BPAL1510-12/265A08	PP600676		Armenia	Gnishik
*P. eriwanensis*	BPAL1468-12/007A07	PP600677		Armenia	Gnishik
*P. eriwanensis*	BPAL1469-12/008A07	PP600678		Armenia	Gnishik
*P. eriwanensis*	BPAL1473-12/019A07	PP600679		Armenia	Gnishik
*P. eriwanensis*	BPAL1474-12/020A07	PP600680		Armenia	Gnishik
*P. keleybaricus* Holotype	BPAL588-11/E262	PP600681	*n* = 86	Iran	Keleybar
*P. keleybaricus*	VL790	PP600682		Iran	Keleybar Makidi
*P. keleybaricus*	VL791	PP600683		Iran	Keleybar Makidi
*P. keleybaricus*	VL792	PP600684		Iran	Keleybar Makidi
*P. ripartii kalashiani*	BPAL1505-12/196A08	PP600685		Armenia	Gyumarants
*P. ripartii kalashiani*	BPAL1506-12/197A08	PP600686		Armenia	Gyumarants
*P. ripartii kalashiani*	BPAL1507-12/202A08	PP600687		Armenia	Gyumarants
*P. ripartii kalashiani*	BPAL1508-12/203A08	PP600688		Armenia	Gyumarants
