# Supplementary figures and images for: Cryptic Taxa Revealed through Combined Analysis of Chromosomes and DNA Barcodes: The Polyommatus ripartii Species Complex in Armenia and NW Iran
Source: Insects. 2024 Jul 19;15(7):545. doi: 10.3390/insects15070545 (PMC11277131; doi:10.3390/insects15070545)

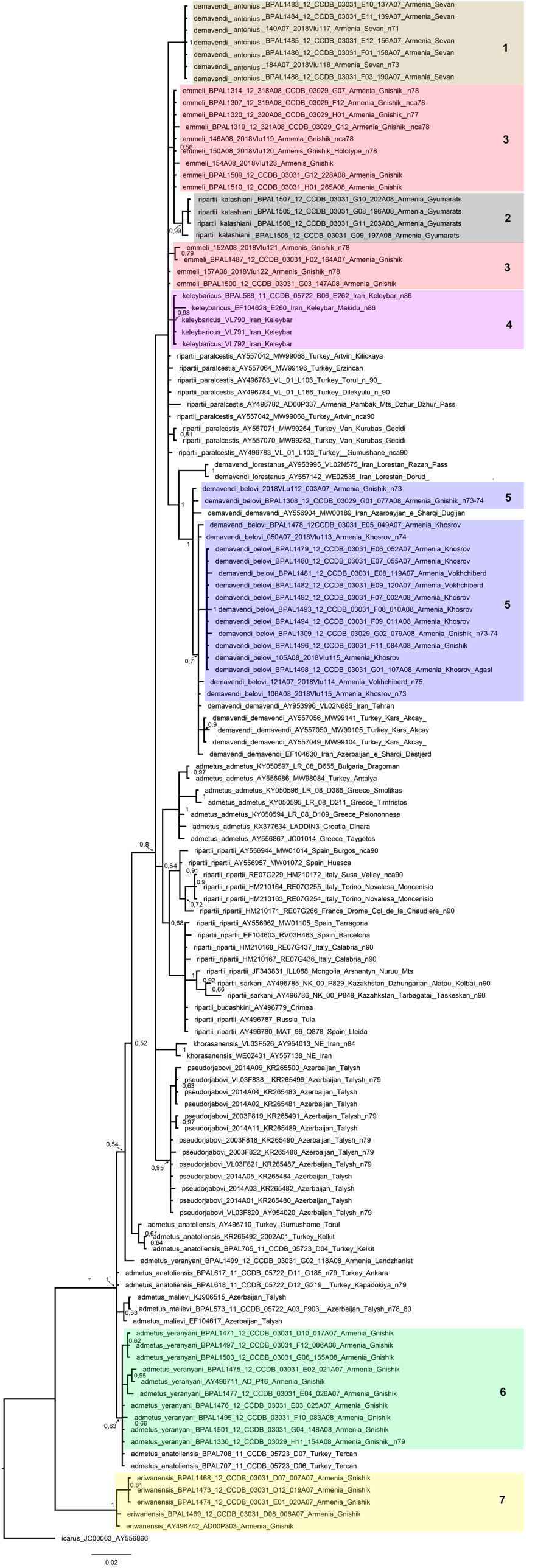

Supplement: Supplementary file 1 [file insects-15-00545-s001.zip › Figure_S1.tif]
